# Influence of Ultrafine Fly Ash and Slag Powder on Microstructure and Properties of Magnesium Potassium Phosphate Cement Paste

**DOI:** 10.3390/ma17112556

**Published:** 2024-05-25

**Authors:** Zheng Jia, Yuhui Zhang, Liwu Mo

**Affiliations:** 1 College of Materials Science and Engineering, Nanjing Tech University, Nanjing 210009, China; jz1473436935@163.com (Z.J.); serenobody@outlook.com (Y.Z.); 2 State Key Laboratory of Materials-Orientated Chemical Engineering, Nanjing 210009, China

**Keywords:** magnesium potassium phosphate cement, ultrafine fly ash, ultrafine slag powder, compressive strength, microstructure, chemically bonded ceramics

## Abstract

This study investigated the influences of ultrafine fly ash (UFA) and ultrafine slag powder (USL) on the compressive strengths, autogenous shrinkage, phase assemblage, and microstructure of magnesium potassium phosphate cement (MKPC). The findings indicate that the aluminosilicate fractions present in both ultrafine fly ash and ultrafine slag participate in the acid–base reaction of the MKPC system, resulting in a preferential formation of irregularly crystalline struvite-K incorporating Al and Si elements or amorphous aluminosilicate phosphate products. UFA addition mitigates early age autogenous shrinkage in MKPC-based materials, whereas USL exacerbates this shrinkage. In terms of the sustained mechanical strength development of the MKPC system, ultrafine fly ash is preferred over ultrafine slag powder. MKPC pastes with ultrafine fly ash show greater compressive strength compared to those with ultrafine slag powder at 180 days due to denser interfaces between the ultrafine fly ash particles and hydration products like struvite-K. The incorporation of 30 wt% ultrafine fly ash enhances compressive strengths across all testing ages.

## 1. Introduction

Magnesium phosphate cement (MPC) is a ceramic type formed via hydration solidification between dead-burned magnesium oxide and phosphate, such as NH_4_H_2_PO_4_ and KH_2_PO_4_ [1]. In the case of MKPC, this hydration process is represented by Equation (1) [2,3]:

MgO(s) + KH_2_PO_4_(s) + H_2_O(l)→MgKPO_4_·6H_2_O(s) (1)

Compared to conventional silicate cement, magnesium phosphate cement offers several advantages, including rapid condensation, great early-age strength, environmental friendliness, and good bonding strength [4,5]. Its applications range widely, including effective fixation of metal contaminants [6], repair grouting [4,7,8,9,10], storage of radioactive waste [11], and biomedical applications [12]. However, MPC faces limitations as a repair material, including the fact that it is non-waterproof, excessive condensation time, and reduced validity under high environmental temperatures or when used in large volumes. In order to enhance MPC properties, mineral admixtures like blast furnace slag [13], fly ash [13,14], calcined kaolin [15], and silica powder [16] are commonly used in MPC paste. However, the impact of mineral admixtures on MPC properties varies based on their variety, phase composition, and additive amount.

In the traditional sense, fly ash was typically viewed as a mere inert material or retarder in the MPC reaction [16]. Its special micromorphology enhances the operability of MPC because it is spherical [8,11]. When combined with silica powder in MKPC, fly ash was reported to enhance water resistance by refining pore structures [16]. Nevertheless, some studies have suggested that adding fly ash decreases MPC’s tensile and residual strength under high temperature curing [17,18]. Recent studies have begun to show that fly ash is more than just a filler in MPC systems [13,19]. Xu et al. [19] used a two-component formula to prepare MKPC mortar mixed with fly ash. When the water–cement ratio was the same, fly ash was regarded as inert or active. In the first recipe, FA was applied as a substitute for two MKPC raw materials (dead-burned magnesium oxide and phosphate) so that the magnesium–phosphorus ratio in the fixed formulation remained constant at 8:1. In another recipe, FA was considered as a substitute only for dead-burned magnesium oxide so that the magnesium–phosphorus ratio in the MKPC changed to 5:1. Due to fly ash’s chemical reaction, replacing MgO alone with fly ash improved the setting time, operability at the end of the stirring, and mechanical properties of MKPC more than replacing both MgO and KH_2_PO_4_ with fly ash [19]. Nicholson and Wilson [20] pointed out that the aluminum–silicon glass phase of FA may hydrate with phosphate after dissolution to produce a gelling material that is conducive to the development of mechanical properties and relatively stable in volume.

The current research indicates that MPC represents a novel gel material characterized by a rapid setting time, high strength, and environmental friendliness. This innovative gel material has found extensive applications in areas such as quick-drying concrete repairs, artificial bone manufacturing, and the treatment of solid waste [21]. Nevertheless, significant challenges persist regarding the practical application of magnesium phosphate cement (MPC), particularly its high preparation cost attributed to the expensive nature of a key ingredient, magnesia. Identifying low-cost alternatives to magnesia beyond conventional magnesite sources is a potential solution to this economic constraint. To mitigate the practical issues associated with MPC, Yang et al. [22] and Li et al. [23] studied the effect of FA on the durability of MKPC in water and salt solutions (sulfate and chloride) and found that the incorporation of fly ash can improve the mechanical properties of MKPC and its pore structure, leading to an improved durability of MKPC with fly ash. Xu et al. [24] found that the addition of FA with a high CaO content, up to 50 wt%, can significantly improve the compressive strength of MKPC. When the fly ash content exceeded 50 wt%, the observed decrease in MKPC strength could be attributed to the formation of CaK_3_H(PO_4_)_2_ and Mg_3_(PO_4_)_2_·22H_2_O. Mo et al. [25] demonstrated that the addition of FA can improve the pore structure of MKPC. Gardner et al. [26] found that FA (or slag) additions at 50 wt% (based on the sum of MgO, KH_2_PO_4_, and H_2_O) resulted in a compact microstructure in the MKPC hardened matrix with an improved mechanical strength at a water-to-solids ratio of 0.24. In summary, incorporating FA can markedly enhance several properties of magnesium potassium phosphate cement (MKPC). These improvements include enhanced workability, mechanical strengths, and durability, alongside a reduction in the heat of hydration and an extension of the setting time. Moreover, employing FA, an industrial by-product, can significantly lower MKPC production costs without adversely affecting its mechanical performance.

With the development of modern civil engineering and environmental engineering, higher requirements have been put forward for the mechanical properties and durability of cementitious materials. Based on this, researchers have developed ultrafine fly ash (UFA) and ultrafine slag powder (USL) using innovative processing techniques on fly ash and slag. UFA is the second superfine grinding product of fly ash, a byproduct from thermal power plants. The ultrafine grinding of fly ash releases the smaller microspheres contained in the larger/hollow fly ash particles [27]. Ultrafine slag powder is the abbreviation of ultrafine granulated blast furnace slag powder, which is a kind of high-quality cement admixture. The industrial solid waste slag obtained from the smelting of pig iron in the blast furnaces of iron plants is mainly composed of calcium silicaluminate as the molten substance. Most of the industrial solid waste slag obtained after water quenching and grinding is glassy and has potential water hard gelling properties. At present, UFA and USL are widely used as mineral admixtures in high performance concrete [28,29]. UFA is an ideal material to utilize due to its smaller particles, which, when mixed with Portland cement, can lead to the formation of ultrahigh-performance concretes [29] and accelerated hydration [30]. These systems were demonstrated to have an improved filling effect associated with the fine particle size of UFA [31], leading to detectable improvements in physical properties, microstructures, and durability [30,32,33]. Similarly, USL, as a common auxiliary hydraulic cementing material, can be incorporated well in the hydration reaction when mixed with Portland cement, thereby improving its performance. In geopolymers, the inclusion of UFA also leads to a reduced porosity [34]. The above studies indicate that UFA could better improve the properties of cementitious materials compared to FA. The performance of solid waste-based MPC is mainly manifested in two aspects: mechanical properties and stability. Current research results have proven that the heavy metal elements in solid waste will result in a decrease in the mechanical properties and stability of MPC in most cases, while the silicon and aluminum elements are able to improve the performance of MPC. Therefore, solid wastes with high silicon and aluminum contents, such as UFA, are appropriate to be solidified and stabilized by MPC. In addition, solid wastes with a low silica–aluminum content and a high content of other elements should be used for a comparative study of specific reaction effects in MKPC.

Therefore, this study investigated the reaction behavior and mechanism in MKPC pastes containing ultrafine fly ash/ultrafine slag powder. The inclusion of ultrafine mineral additives, especially at higher levels, can alter the reaction conditions (such as acidity and alkalinity, degree of hydration, ion mobility, etc.) within the MKPC system, potentially affecting its reaction behavior, microstructure formation, and subsequent strength development. This study seeks to explore how ultrafine fly ash and ultrafine slag powder impact the microstructure, compressive strength, and volume stability of MKPC pastes at five different substitution levels: 0 wt%, 20 wt%, 30 wt%, 40 wt%, and 50 wt% of the total MgO weight. Additionally, we examined the potential chemical reactions of ultrafine fly ash and ultrafine slag powder within the same MKPC paste system using XRD, TG/DSC, and SEM/EDS. Moreover, we measured the early volumetric self-shrinkage properties of MKPC mortar for the first time using a German Micro-Epsilon displacement sensor (MICRO-EPSILON MESSTECHNIK GmbH & Co. KG, Ortenburg, Germany), which has not been widely used in this field before. This also opens up new research perspectives and provides a more direct and reliable method for future researchers. This research aims to offer foundational insights into the properties of MKPC altered by ultrafine fly ash or ultrafine slag powder, along with their associated mechanisms, which is valuable for advancing the properties of MKPC.

## 2. Experimental

### 2.1. MKPC Paste Preparation

**Figure 1:**
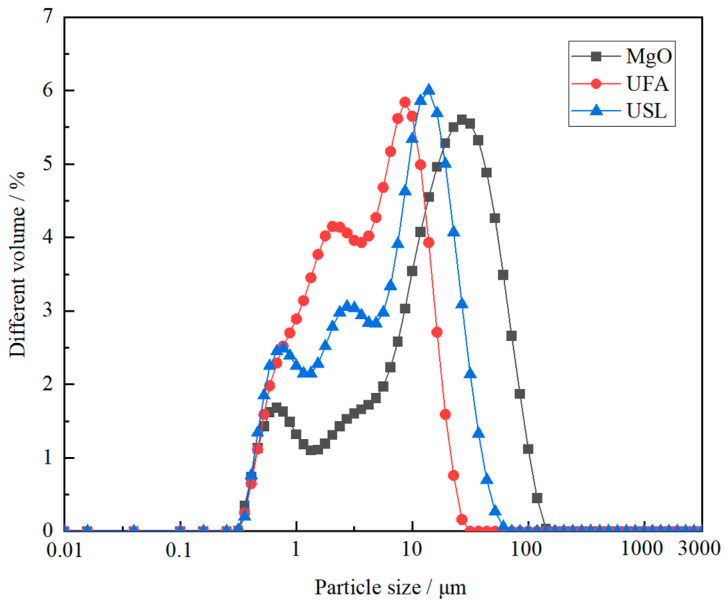
Particle size distributions of MgO, ultrafine fly ash, and ultrafine slag powder.

Yancheng Hua Nai Magnesium Industry Co., Ltd., Yancheng, China, provided the dead-burned MgO, which was obtained by calcining magnesite at 1500 °C. Shi fang Dingli Co., Ltd. in Sichuan, China supplied industrial-grade KH_2_PO_4_. Sodium tetraborate was added as the solidification retarder in a constant amount of 10.0 wt% relative to the weight of MgO. Ultrafine fly ash and ultrafine slag powder were used to partially substitute MgO. The chemical compositions of MgO, ultrafine fly ash, and ultrafine slag powder are outlined in Table 1. Specifically, the ultrafine slag powder contains 11.89 wt% Al_2_O_3_, 33.39 wt% SiO_2_, and 41.51 wt% CaO. In contrast, the ultrafine fly ash has higher levels of Al_2_O_3_ and SiO_2_, at 31.65 wt% and 50.53 wt%, respectively. Al_2_O_3_ and SiO_2_ in the ultrafine slag powder are primarily present as amorphous aluminosilicate, while in the ultrafine fly ash, they exist as mullite crystalline phases and a glassy aluminosilicate phase. Additionally, a portion of quartz is present in the ultrafine fly ash. The ultrafine fly ash and ultrafine slag powder used in this paper are in accordance with T/CBMF194-2022 [35] China Building Materials Association standard industry document specifications. Particle size distributions of dead-burned magnesium oxide, ultrafine fly ash, and ultrafine slag powder were analyzed using a powder laser particle size analyzer, as displayed in Figure 1. 

The mechanical properties of MPC are significantly affected by the M/P ratio [36,37]. Previous research has shown that the best mechanical performance of MKPC is achieved with an M/P molar ratio of 3~4:1 [38,39]. In this study, the control mix had an initial magnesium-to-phosphorus ratio of 3:1. Nine sets of MKPC were prepped, with ultrafine fly ash and ultrafine slag powder utilized as partial substitutes for MgO alongside the control. Table 2 details the composition of alkaline components in MKPC. Various levels of substitution of MgO with ultrafine fly ash and ultrafine slag powder were tested, ranging from 20 wt% to 50 wt%. The water–cement ratio remained at 0.18. Before adding water, the powdered raw materials (including magnesium oxide, KH_2_PO_4_, sodium tetraborate, UFA, and USL) were pre-blended to ensure uniformity.

Then, water was added to the MKPC mixture and mixed continuously until a homogeneous mixture was achieved. The resulting mixture was poured into a 40 mm × 40 mm × 40 mm mold to form a cubic mortar at a temperature of 20 ± 2 °C and a relative humidity (RH) of 50 ± 5% in a laboratory environment. The mold was covered with plastic film and left to stand for 2 h. The specimens were then removed from the molds and cured at room temperature for different times: 2 h, 24 h, 28 days, and 180 days.

### 2.2. Compressive Strength Test

The compressive strengths of MKPC-bonded specimens were evaluated in multiple stages, specifically at 2 h, 24 h, 28 days, and 180 days. For each examination, an average compressive strength value was derived from five individual paste cubes.

### 2.3. Autogenous Shrinkage Test

The German Micro-Epsilon displacement sensor was used to test the early volume shrinkage of the mortar. The length of the threaded pipe was 42 cm, the outer diameter was 28 mm, and the inner diameter was 20 mm. One end of the threaded pipe was fixed and the sensor probe was adjusted so that the shrinkage or expansion of the mortar was within the measuring range of the instrument. During the test, the threaded tube device and laser equipment were kept stable, and the ambient temperature was controlled at 20 ± 3 °C.

### 2.4. Mineral Phase Examination

The required samples were examined using an X-ray diffractometer. The samples were prepared using the powder diffraction method by first drying the specimens at 50 °C, then grinding them to less than 80 μm using an agate mortar and pestle, and then placing the fine powder into a 0.5 mm-deep glass sample stage and slightly compacting the powder surface using a smooth glass sheet. For XRD measurements, the device was set to work with a target of Cu Kα, a tube current of 100 mA, and a tube voltage of 40 kV. The scanning was carried out with a speed setting of 10°/min and covered an angular range of 5° to 60°.

### 2.5. Pore Structure Test

The pore composition within set MKPC was meticulously examined utilizing a Pore Master GT-60 Mercury Intrusion Porosimeter (MIP). To prepare for this analysis, the solidified MKPC paste was fragmented into diminutive pieces ranging from 2 to 5 mm. These fragments were then submerged in pure ethyl alcohol to cease hydration processes, followed by a vacuum drying process at a temperature of 45 °C to ensure complete dryness prior to the pore size evaluation.

### 2.6. Microstructure Examination Using SEM

A selected sample from a newly damaged area of the cured MKPC specimen was soaked in pure ethanol to stop continued reaction. After halting hydration, the sample was dried at 50 °C and thinly coated with gold. The microstructural characteristics and composition of emerging compounds within the MKPC pastes were examined using an LEO 1530VP Scanning Electron Microscope (SEM) (Zeiss AG, Baden-Württemberg, Germany) equipped with an Oxford Energy Dispersive Spectroscopy (EDS) system.

Moreover, for further examination, a cubic piece of the set MKPC paste was sectioned into thin slices, underwent a similar cessation of hydration in absolute ethyl alcohol, and was then vacuum-dried. The arid specimen was infused with an epoxy resin, polished to achieve a smooth surface, coated with carbon to make it conductive, and analyzed with the SEM operated in the backscattered electron (BSE) mode for high-contrast imaging. Concurrently, the EDS was used to carry out a precise elemental analysis of selected areas of interest.

## 3. Results and Discussion

### 3.1. Phase Assemblage in MKPC Pastes

**Figure 2:**
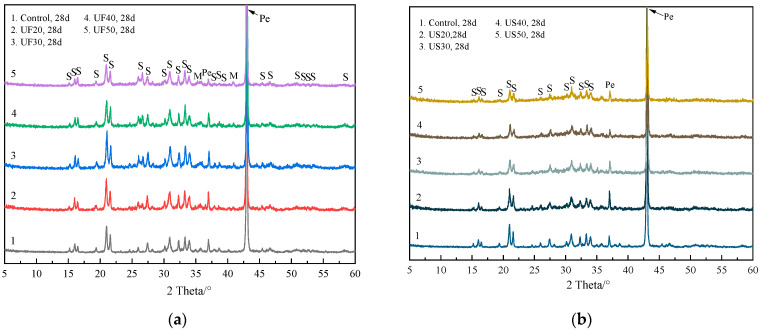
Powder diffraction patterns for MKPC pastes at 28 days of curing: (**a**) mixed with ultrafine fly ash and (**b**) mixed with ultrafine slag powder. Pe: periclase, S: struvite-K, Q: quartz, M: mullite.

Figure 2 presents XRD spectra of MKPC pastes at 28 d with different doses of ultrafine fly ash and ultrafine slag powder. Regardless of the presence of ultrafine fly ash or ultrafine slag powder, struvite-K was still the primary crystalline hydration product in all MKPC specimens, as shown in the figure. No new crystalline phases were detected as the curing period extended to 28 d, indicating the complete reaction of KH_2_PO_4_ upon the addition of ultrafine fly ash or ultrafine slag powder. Across all MKPC pastes, abundant unreacted MgO was evident in all test samples, as indicated by XRD spectra. Similarly, the inclusion of ultrafine fly ash and ultrafine slag powder did not lead to the formation of novel crystalline products compared to the control sample.

**Figure 3:**
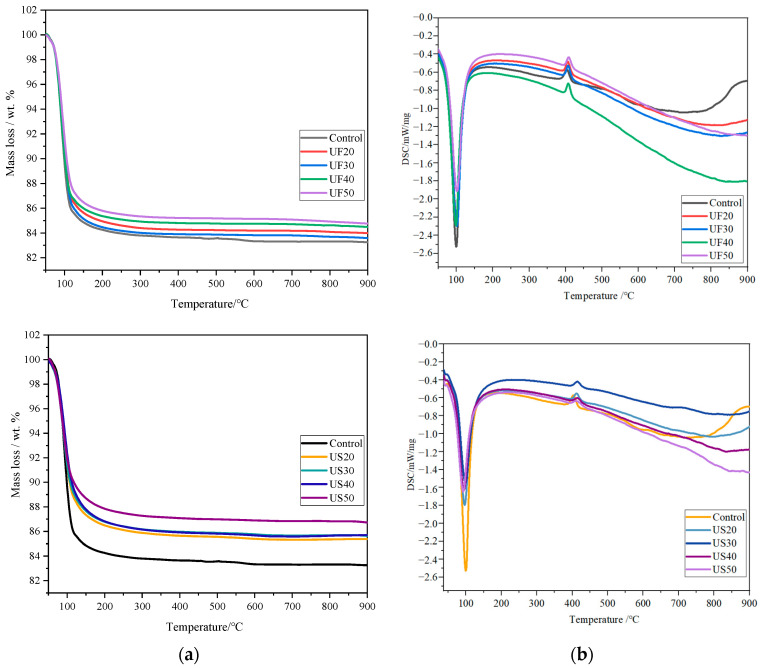
TG/DSC curves for MKPC pastes with ultrafine fly ash or ultrafine slag powder (28 d). (**a**) TG curves and (**b**) DSC curves.

Figure 3 illustrates TG/DSC curves at 28 d for MKPC pastes with additions of ultrafine fly ash or ultrafine slag powder. Endothermic peaks can be observed around 100 °C in all MKPC pastes, attributed to dehydration of hydration products like struvite-K. Within the temperature range of 60–150 °C, MKPC paste mass losses were 14.4 wt%, 14.9 wt%, 13.6 wt%, 12.6 wt%, and 12.2 wt% for UF20, UF30, UF50, US20, and US40, respectively, primarily due to struvite-K dehydration. Consequently, adding 30 wt% ultrafine fly ash slightly increased the struvite-K quantity compared to UF20 paste, but UF50 showed lower mass loss than UF30. Substituting MgO with 30 wt% UFA increased the water for cement reaction owing to dilution, facilitating the reaction somewhat. However, it reduced MgO and KH_2_PO_4_ quantities, potentially decreasing the overall struvite-K quantity. For UF50, decreased total MgO and KH_2_PO_4_ quantities might mainly reduce the struvite-K quantity. Unlike in MKPC pastes with ultrafine slag powder, the struvite-K quantity gradually decreased with the increasing dosage. Exothermic peaks around 420 °C can be observed in MKPC pastes with ultrafine fly ash or ultrafine slag powder, albeit with no obvious mass losses (Figure 3a). Further investigation is needed to understand this phenomenon. No endothermic peak indicating KH_2_PO_4_ dehydration, typically occurring within 200–300 °C, was observed, indicating the absence of KH_2_PO_4_ in the pastes, consistent with XRD analysis.

### 3.2. Compressive Strength

**Figure 4:**
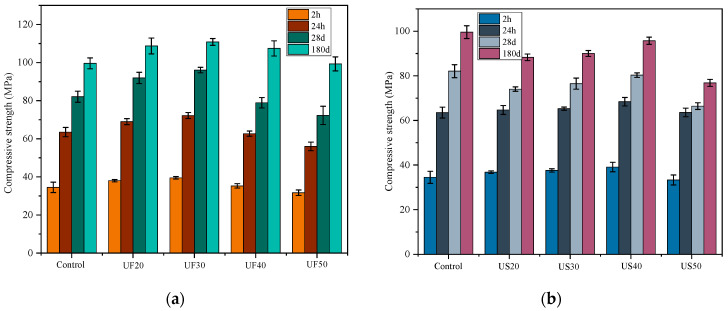
Compressive strengths of the MKPC pastes containing different doses of (**a**) ultrafine fly ash and (**b**) ultrafine slag powder.

Figure 4 illustrates the compressive strengths of MKPC mortars at various time points: 2 h, 24 h, 28 days, and 180 days. The compressive strength of the control mortar was 34.5 MPa at 2 h, rising to 82.1 MPa and 99.6 MPa at 28 days and 180 days, respectively. UF30 displayed notably higher compressive strengths compared to the control mixture at the same age. As shown in Figure 4a, at 180 days, the compressive strengths of the control, UF20, UF30, UF40, and UF50 were 99.6 MPa, 108.7 MPa, 110.9 MPa, 107.4 MPa, and 99.3 MPa, respectively. However, ultrafine slag powder exhibited varied effects on the compressive strengths of MKPC. For example, US20, US30, and US40 showed higher compressive strengths than the control at the early stage of 2 h (Figure 4b), but they did not progress as well as the control at later stages (180 d). The increased addition of ultrafine fly ash in MKPC mortar from 30 wt% to 50 wt% led to a slight decline in compressive strength in the early stages of curing at 2 h, 24 h, and 28 days. Nevertheless, at 180 days, UF50 achieved a compressive strength proximate to the control. Compared to MKPC containing ultrafine slag powder, MKPC containing ultrafine fly ash demonstrated superior strength development overall. For example, the compressive strengths of UF30 at 2 h, 28 days, and 180 days were 39.5 MPa, 96.1 MPa, and 110.9 MPa, respectively, higher than the corresponding 37.6 MPa, 76.5 MPa, and 90.0 MPa of US30. Furthermore, at the higher incorporation rate of 50 wt%, more significant variations were observed between the compressive strengths of MKPC mortar with ultrafine fly ash and that mixed with ultrafine slag powder at 180 days. This indicates that ultrafine fly ash contributes more to the long-term compressive strength development of MKPC mortar compared to ultrafine slag powder.

### 3.3. Autogenous Shrinkage

**Figure 5:**
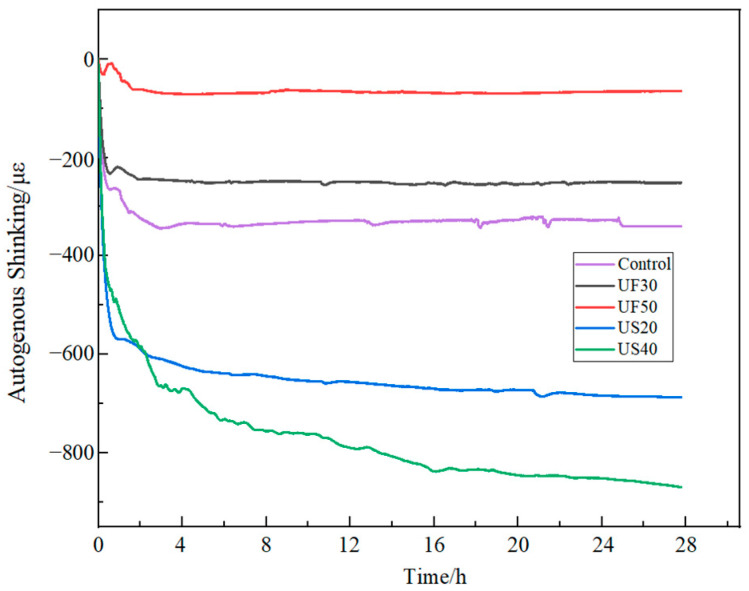
Autogenous shrinkage of MKPC mortar mixed with ultrafine fly ash or ultrafine slag powder.

In this study, the early volume autogenous shrinkage of MKPC mortar was examined using a Micro-Epsilon displacement sensor from Germany. Figure 5 illustrates the impact of ultrafine fly ash and ultrafine slag powder on the autogenous shrinkage of MKPC mortar. It is evident from Figure 5 that the incorporation of ultrafine fly ash has a positive effect on the early volume autogenous shrinkage of magnesium phosphate cement-based materials. The final volume autogenous shrinkage of the UF30 mortar was 251 με, which represents a 26% reduction compared to the control group mortar at 339 με. More notably, the MKPC mortar with 50 wt% ultrafine fly ash (UF50) exhibited a final volume autogenous shrinkage of 65 με, achieving an 80.8% decrease compared to the final volume autogenous shrinkage of the control group mortar. In contrast, the addition of ultrafine slag powder demonstrated a different effect by increasing the early autogenous shrinkage of the magnesium phosphate cement mortar. For instance, compared to the control group mortar, the final volume autogenous shrinkage of the US20 mortar increased by 348 με. This phenomenon may be associated with the consumption of retarders due to the addition of ultrafine slag powder. In summary, the incorporation of ultrafine fly ash is more beneficial for improving the early volume stability of MKPC cement mortar.

### 3.4. Microstructure

#### 3.4.1. Pore Structure Analysis

**Figure 6:**
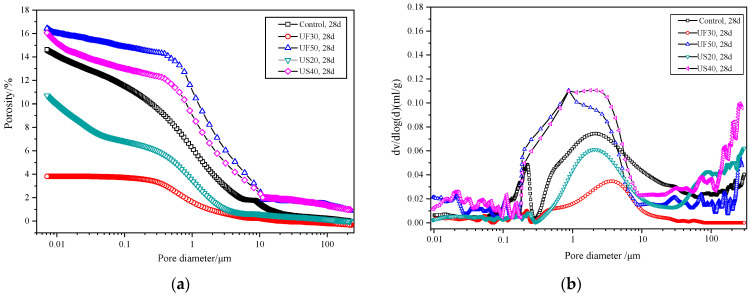
Pore structure analysis plots of MKPC pastes with or without the addition of ultrafine fly ash/ultrafine slag powder: (**a**) porosity and (**b**) pore size distribution.

Figure 6 depicts the pore structures of MKPC pastes with and without the inclusion of ultrafine fly ash/ultrafine slag powder. The addition of 30 wt% ultrafine fly ash (UF30) resulted in a decrease in the volume of pores with diameters ranging from 0.1 to 10 μm at 28 days compared to the control (Figure 6b). However, similar pore structures between UF50 and US40 were observed within the 0.1 to 10 μm range at 28 days. With an increase in the addition of ultrafine fly ash to 50 wt% (UF50), there was a notable increase in the volume of pores with diameters larger than 0.1 μm, leading to a higher total porosity compared to the control and UF30.

The addition of ultrafine slag powder in moderate amounts had a similar effect on the pore microstructure of MKPC as the incorporation of ultrafine fly ash. US20 showed fewer pores with diameters from 0.1 μm to 10 μm and a smaller porosity of 10.7% at 28 days compared to the control. In comparison to MKPC containing ultrafine slag powder, MKPC supplemented with ultrafine fly ash had a visibly denser structure with less porosity, especially at the 30 wt% addition. For example, the total porosity of UF30 was 4.2%, which is less than the total porosity of 10.7% for US20 at 28 days (Figure 6a).

#### 3.4.2. Morphology

**Figure 7:**
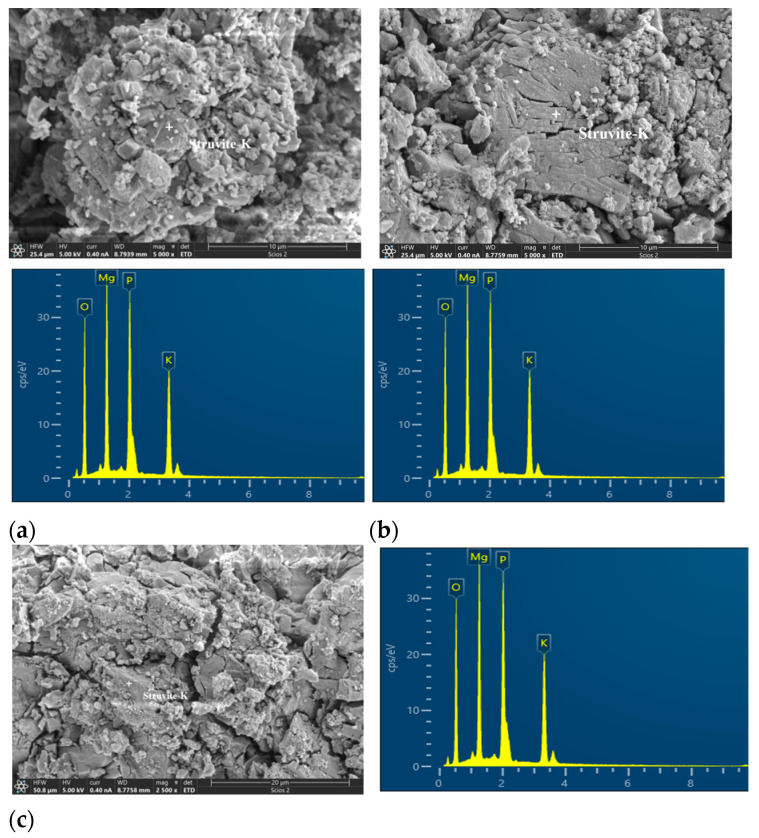
SEM microscopy morphology of the control group at 28 d: (**a**) loosely agglomerated sheeted struvite-K crystals, (**b**) compact columnar struvite-K crystals, (**c**) irregularly shaped struvite-K. Note: “+” is the location of the DES scanning point.

Figure 7 displays the typical struvite-K morphology observed in the control paste. The formation location heavily influences the morphology of struvite-K. In porous and loose regions, struvite-K generally forms sizable tissues (Figure 7a). Large lamellar structures (Figure 7a) and laminated ribbed particles (Figure 7b) are visible within pores, as depicted in Figure 7, consistent with previous findings indicating struvite-K’s propensity to grow larger in open spaces like pores [40]. In contrast, in closed regions, irregular but tightly packed crystals emerge (Figure 7c). Ding et al. [34] identified two common forms of struvite-K in solidified paste: crystalline and amorphous phases. MKPC paste reaction products exhibit visible cracks. Previous studies attributed cracks to potential product dehydration under a vacuum [41]. However, Ma et al. [42] proposed that cracks represent gaps between portions of attached struvite-K particles because vacuum drying at room temperature does not dehydrate struvite-K. Sample processing, such as fracturing and vacuum drying, may induce micro-cracks. Furthermore, our research indicates that product gaps appear in large crystals with regular shapes (e.g., as depicted in Figure 7a,b), resembling cracks under SEM observations, especially in BSE mode.

**Figure 8:**
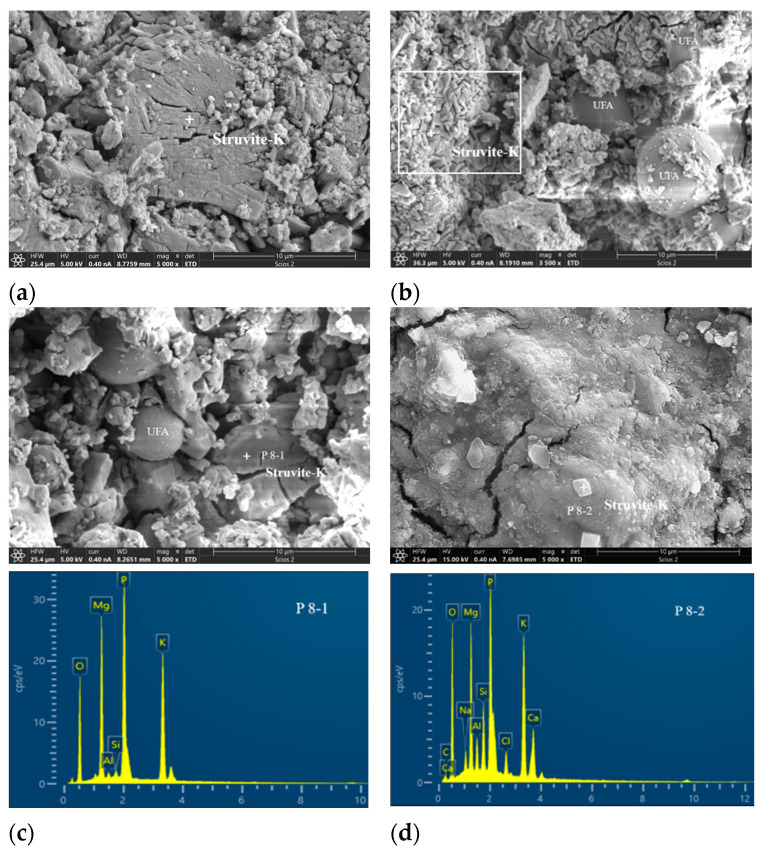
SEM images of MKPC pastes with and without ultrafine fly ash or ultrafine slag powder: (**a**) control, (**b**,**c**) UF30, (**d**) US40. Note: “+” is the location of the DES scanning point, and the white area is the significant accumulation area of struvite-K.

Figure 8 showcases scanning electron microscope pictures of MKPC containing either ultrafine fly ash or ultrafine slag powder. In a manner akin to the control, a number of large lamellar structures formed within a pore in the UF30 sample (Figure 8a). Far from pores, distinct ultrafine fly ash pellets with a spherical shape were embedded in the solidified UF30 paste (Figure 8b). Careful observation of the region delineated by a white square revealed irregular struvite-K formation, contributing to a denser microstructure compared to the control paste. Trace amounts of Si and Al elements were discernible in the EDS spectra at spot P8-1 (Figure 8c), suggesting the integration of Si and Al elements into products. There were also some cracks in the reaction product of the MKPC paste containing ultrafine fly ash, but the number of cracks was significantly lower than in the control sample.

In the MKPC incorporating ultrafine slag powder, depicted in Figure 8d, lumpy reaction products of variable shapes developed surrounding particles of ultrafine slag powder. Based on the EDS energy spectrum at spot P8-2, these reaction phases comprised struvite-K with certain levels of Si and Al (Figure 8d). Likewise, as illustrated in Figure 8e, layered mass crystals formed around particles of ultrafine slag powder, also recognized as struvite-K, yet showing more pronounced peak intensities of Si, Al, and even Ca in the EDS spectrum at hydration spot P8-3. It is worth noting that with the addition of ultrafine fly ash or ultrafine slag powder, the resultant reaction product, struvite-K, exhibited irregular phase shapes, a rough interface, and bulges in comparison with the control. This occurrence could be attributed to the integration of Al and Si elements into the struvite-K crystals or the presence of fresh aluminum silicate potassium phosphate crystals [13].

**Figure 9:**
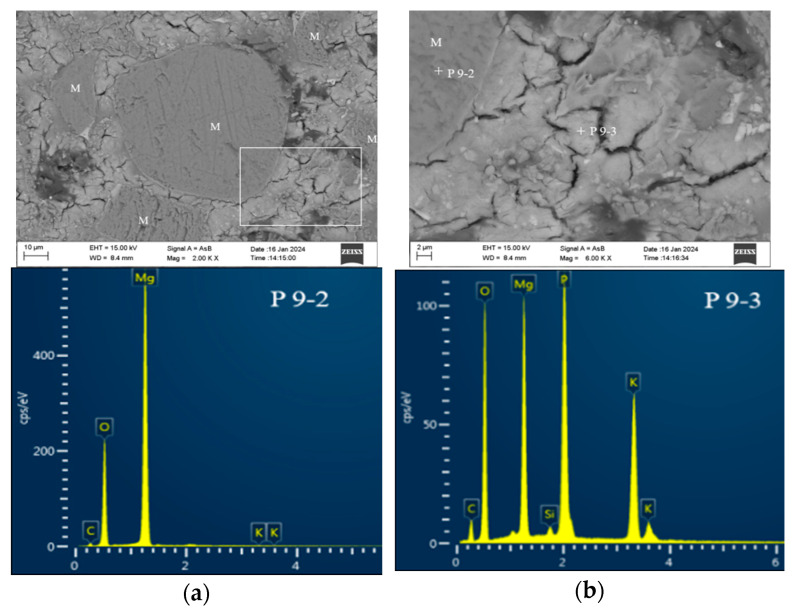
BSE images of the control paste: (**a**) low magnification, (**b**) high magnification, and close observation of the white square-marked area in (**a**). Note: “+” is the position of the DES scanning point, and the white area is the enlarged observation area.

Figure 9 displays a representative backscatter picture of the reference. The solidified MKPC was composed of struvite-K and numerous unreacted magnesia particles. Large prismatic crystals, demonstrating a large prismatic structure, were commonly located in close proximity to pores or within porous areas (Figure 9b). In the central portion of Figure 9a, larger aggregates of crystals with fissures are noticeable. Struvite-K crystals developed around the columnar magnesium oxide fractions. However, it appears that not all magnesium oxide fractions were effectively bound to reaction products due to noticeable gaps between the original blended cement and the MgO pellets. Several fissures were evident in the paste, aligning with the observations from SEM analyses.

**Figure 10:**
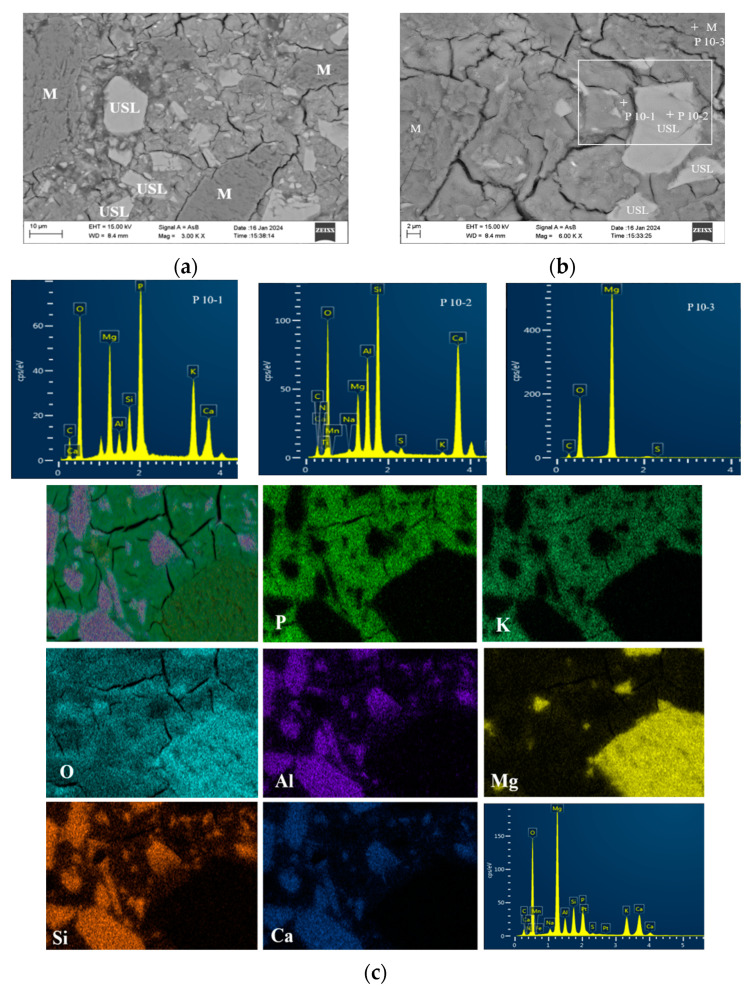
BSE images of the MKPC with ultrafine slag powder: (**a**) paste morphology, (**b**) paste with higher magnification, and (**c**) backscatter surface scanning images and elemental maps. Note: “+” is the position of DES scanning point, and the white area is the interface area between USL and paste.

Figure 10 showcases standard BSE images of MKPC pastes incorporating ultrafine slag powder. As depicted in Figure 10a, particles of ultrafine slag powder were distributed across the MKPC paste, leading to a porous microstructure with many cracks, in contrast to UF30. A detailed inspection of the area indicated by a white square unveiled the creation of compact hydration products encasing the ultrafine slag powder particles (Figure 10b). EDS examination at point P10-1 confirmed the existence of Al, Si, and Ca elements in the product, aligning with the SEM analysis.

**Figure 11:**
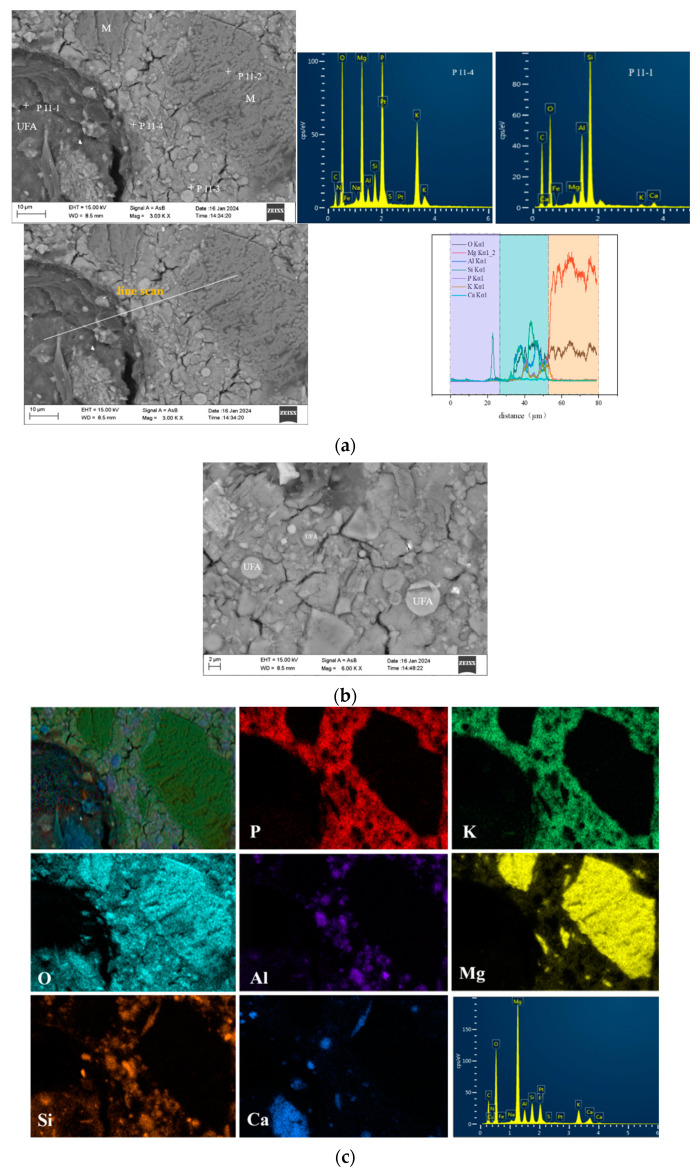
BSE images of the MKPC paste with ultrafine fly ash: (**a**) UF30, (**b**) UF30 with higher magnification, and (**c**) backscatter surface scanning images and elemental maps of UF30. Note: “+” is the position of DES scanning point.

Figure 11b exhibits an MKPC paste with 30 wt% ultrafine fly ash at 28 days. Ultrafine fly ash particles were embedded in the UF30 paste matrix, resulting in a denser paste with fewer cracks compared to the control and US20 pastes (Figure 9 and Figure 10). Moreover, distinct hydration product rims encased the ultrafine fly ash particles, establishing a significantly denser surface between the ultrafine fly ash fraction and the original cement (Figure 11a). EDS analysis (Figure 11a) indicated that the hydration products were composed of the elements Mg, O, P, K, Si, and Al, suggesting that silicon and aluminum were integrated in the structure, consistent with the observations in Figure 10. Gardner et al. [13] also noted the integration of Al and Si into the combined substrate of MKPC incorporating FA based on BSEM images and elemental plots. This integration was attributed to the solubilization of the glassy portion of aluminosilicates in the FA, leading to the creation of an aluminosilicate potassium phosphate-binding phase [13]. Secondary electron microscopy demonstrated that struvite-K crystals effectively encapsulated the ultrafine fly ash pellets, indicating favorable tissue compatibility between them [19]. Nonetheless, a number of ultrafine fly ash particles exhibited noticeable gaps at the boundary with struvite-K. Within the UF30 paste, certain MgO particles remained unreacted. As can be observed in Figure 11b, needle-like or bulk phases were interspersed with amorphous struvite-K, creating an intensive blend microstructure. The hybrid phase may represent an aluminosilicate-rich product. This blend structure is presumed to enhance strength.

### 3.5. Discussion

#### 3.5.1. Physicochemical Effects of Ultrafine Fly Ash and Ultrafine Slag Powder in MKPC System

The reactions of ultrafine fly ash and ultrafine slag powder in MKPC have both physical and chemical effects. Physically, the incorporation of ultrafine fly ash or ultrafine slag powder has a diluting impact on the MKPC paste. In this study, the addition of a high volume of ultrafine admixture actually increased the amount of water reacting between MgO and KH_2_PO_4_ due to the fixed water–cement ratio. Despite incorporating ultrafine fly ash instead of raw materials, the quantities of reaction products, particularly struvite-K, appeared similar or showed only a slight difference (as indicated by XRD and DSC/TG) compared to those of the control. This suggests that MKPC pastes with UFA achieved a great degree of reaction. Given the constant water–cement ratio, the inclusion of UFA or USL may impact pH levels and consequently affect the hydration. According to Rouzic et al. [29], the kind of hydration product in MKPC systems heavily relies on the acidity and alkalinity. For example, newberyite is dominant in a pH range of four to six, a combination of newberyite and struvite-K typically forms at pH six to seven, and struvite-K becomes the primary product when the pH exceeds seven. Furthermore, the dispersion of ultrafine fly ash or slag powder particles within MKPC pastes provides nucleation sites for struvite-K precipitation, thereby influencing the position distribution of reaction products.

Regarding the chemical points, both UFA and USL participate in the reaction process, albeit to varying degrees. When either UFA or USL was added, as observed in X-ray diffraction (XRD) patterns, no other product was formed in MKPC apart from struvite-K. However, scanning electron microscopy (SEM) coupled with energy-dispersive X-ray spectroscopy (EDS) reveals that in the presence of either UFA or USL, a number of Si and Al elements are present in the hydration product, struvite-K. Gardner et al. [26] discovered through microstructure characterization and multi-nuclear NMR spectra that the aluminosilicate glassy fractions of both fly ash and GBFS dissolve within the MKPC binders under near-neutral pH conditions and subsequently form second-reactive species rich in silicon and aluminum. This has the potential to form an aluminosilicate potassium phosphate phase.

In our study, binding products containing Si and Al were also observed in the MKPC pastes with either UFA or USL (Figure 10 and Figure 11). This phenomenon suggests that Si and Al elements are bound to main hydration products. Additionally, amorphous hydration products, such as the potassium aluminosilicate phosphate phase, might form due to the geopolymer synthesis of soluble aluminum silicate species. However, owing to their amorphous character, they cannot be analyzed via X-ray diffraction. Apart from aluminum, it has been reported that other components of FA, particularly magnesium and calcium, compete against magnesium oxide and react with KH_2_PO_4_, forming phosphate-containing colloids, thereby altering the initially designed M/P molar ratios and potentially enhancing mechanical properties [19]. A fraction of magnesium oxide in FA is anticipated to contribute to the formation of struvite-K [19]. The calcium component of FA also hydrates with KH_2_PO_4_ to form an amorphous calcium phosphate product [43]. Ma et al. [42] proposed that synergistic effects between GBFS and MKPC might largely be due to the fact that CaO in GBFS reacts with KH_2_PO_4_ to create calcium phosphate, given that GBFS contains a higher CaO content and exhibits better mechanical properties. Nevertheless, our study appears to contradict this assumption, since ultrafine fly ash has a lower content of calcium oxide but still achieved better mechanical properties.

Alumina oxide demonstrates reactivity and can potentially result in the formation of magnesium alumina phosphate in MPC [44]. As elaborated by Wagh [44], in a phosphate solution, alumina dissolves and reacts with phosphate ions in solution to produce AlH_3_(PO_4_)_2_·H_2_O, which then further reacts with the remaining alumina to yield the ultimate product of AlPO_4_. It is recognized as an amorphous gel-like tissue that can form under ambient temperatures [44,45,46] or at elevated temperatures ranging from 50 to 100 °C [47]. From the structural analysis of the products, substituting a fraction of the magnesium atoms with aluminum possibly results in a material resembling a geopolymer within the MPC matrix [48,49]. Ultrafine fly ash demonstrates higher chemical instability compared to alumina, primarily attributable to the presence of its glassy aluminosilicate phase and the structural disruption induced by milling. The envisaged reaction mechanism suggests that, within the acidic or nearly neutral conditions of the MKPC environment, Al could leach from ultrafine fly ash, likely in an aluminosilicate phase. Subsequently, the Al-rich entities could serve as attachment sites or furnish crystallization sites for the deposition of K^+^, Mg^2+^, and PO_4_^3−^ ions, culminating in the formation of struvite-K.

The factors affecting the solubilization rate of aluminum silicate in slag, fly ash, and other admixtures containing silica–alumina phases have been reported, including the temperature, alkalinity, crystallinity, dissolution type, and structural defects [50,51]. Due to the high content of the aluminosilicate glass phase in ultrafine fly ash, it exhibits an elevated rate of dissolution in the acidic or nearly neutral milieu of the magnesium potassium phosphate cement (MKPC) system. Within this system, ultrafine fly ash demonstrates enhanced reactivity, potentially resulting in reaction products such as struvite-K containing increased levels of aluminum and silicon. Given the significant role of pH levels, our forthcoming research endeavors will delve into the dissolution behaviors of ultrafine fly ash and slag powder within MKPC systems.

#### 3.5.2. Influence of Ultrafine Fly Ash or Ultrafine Slag Powder on the Microstructure of MKPC

The integration of ultrafine fly ash and ultrafine slag powder into the MKPC matrix forms a ternary composite structure. This structure comprises the unreacted magnesium oxide (MgO), the ultrafine constituents (fly ash/slag powder), and the hydration products, notably amorphous aluminosilicate phosphate and struvite-K. The incorporation of these ultrafine materials modifies the positional distribution of other amorphous reaction products and struvite-K. These products precipitate around the MgO particles and the ultrafine components, fostering a cohesive matrix by connecting these particles. Incorporating 30 wt% of ultrafine fly ash or 20 wt% ultrafine slag powder into the MKPC paste leads to a reduction in pore size and overall porosity, enhancing the material’s density. Conversely, increasing the content of these ultrafine materials beyond these proportions to 40 wt% or 50 wt% inversely affects the paste’s total porosity, making it more porous. Specifically, comparing compositions with 30 wt% ultrafine fly ash (UFA) and 20 wt% ultrafine slag powder (USL), the paste containing 30 wt% UFA showcases a more compact microstructure compared to the 20 wt% USL variant. This densification correlates with a notable decrease in total porosity, especially in the reduction in pores within the 0.1 μm to 10 μm range, likely due to the enhanced reactivity of ultrafine fly ash within the MKPC pastes. An interesting observation is the decreased incidence of cracks within the MKPC paste matrix following the addition of ultrafine fly ash. This phenomenon could be attributed to the augmented strength and improved interconnectivity among the amorphous reaction products, which contribute to the structural integrity of the paste.

The microstructural characteristics of struvite-K are fundamental to the mechanical performance, transport characteristics, and longevity of magnesium potassium phosphate cement (MKPC). The present study observes that struvite-K within MKPC pastes manifests in two distinct forms: crystalline and amorphous. The occurrence of these forms is contingent upon the specific precipitation sites within the matrix. Crystalline struvite-K is typically precipitated within pores or porous areas, while amorphous struvite-K forms in denser or more constrained spaces. The formation of voids within MKPC pastes is predominantly a result of water evaporation during the reaction process, with higher water contents leading to an increased void volume. Consequently, the water content critically influences the crystalline structure and the morphological traits of the hydration products in magnesium phosphate cement (MPC) mortar. Supporting evidence can be found in the literature [52], where an elevation in water content from 5 wt% to 8 wt% was observed to correlate with the augmented formation of crystalline magnesium ammonium phosphate hexahydrate. It is worth noting that the morphological and structural attributes of the struvite-K crystals are key indicators of the MKPC’s physical properties. The delicate balance between crystalline and amorphous phases, driven by the water content in the mix, plays a pivotal role in optimizing the material’s attributes for enhanced durability and performance.

Previous studies have reported that the morphological characteristics of struvite-K are influenced by the magnesium–phosphorus ratio, as well as the acidity or alkalinity of the solution [36,53,54]. Specifically, at a magnesium–phosphorus ratio of 3, struvite-K was found to crystallize into a mixture of needle-like and plate-like forms. When the ratio was elevated to 5, the crystalline structure transitioned into a distinctly prismatic configuration. However, at a Mg–P ratio of 10, the resulting struvite-K was characterized by an indistinct shape [36]. Chau et al. [55] observed that a pH value of 6 fostered the formation of struvite-K crystals with a plate-like morphology, while at a pH of 7.5, prismatic crystals were predominant. A converse relationship between pH and crystal size was noted, with smaller struvite-K crystals forming as the pH diminished [56]. However, our study discovered that the addition of UFA or USL promoted the genesis of amorphous products. This suggests that the involvement of silicon and aluminum ions during the deposition process significantly alters the conventional crystalline structure of struvite-K.

#### 3.5.3. Effect of Ultrafine Fly Ash or Ultrafine Slag Powder on the Mechanical Properties and Volume Stability of MKPC

In the case of MKPC, the presence of both unreacted dead-burned magnesium oxide and the hydration product forms a sturdy substrate, contributing to its strength [57]. The unreacted MgO plays the role of an adsorption aggregate within the paste, thereby helping to improve its mechanical properties [57,58]. Considering the excellent surface properties of calcined magnesium oxide, the mechanical strength can be comprehensively improved by increasing proportion of MgO, i.e., increasing the magnesium oxide content in the MPC [36]. Nevertheless, there is an optimal ratio of unreacted MgO “hard core” to struvite “binder” to achieve optimal mechanical properties [4,36]. Beyond this optimal ratio, increasing the initial MgO content may actually decrease the compressive strength because of reduced hydraulicities [36]. It has also been argued that the strength of MKPC pastes is largely derived from the bond strength between unreacted dead-burned magnesium oxide and the hydration product [58]. In our study, there is still an observable excess of unreacted MgO in the MKPC pastes compared to KH_2_PO_4_ due to its stoichiometry. However, there appeared to be insufficient binding between the MgO and the struvite-K, as a clear gap was observed on the SEM. Similar to the unreacted magnesium oxide, the remaining ultrafine fly ash/ultrafine slag powder can also serve as a filler in the MKPC matrix. Moreover, higher dosages of ultrafine fly ash/ultrafine slag powder in MKPC result in decreased residual magnesium oxide. The ultrafine doped particles are softer than the dead-burned MgO particles, leading to reduced bonding between unreacted MgO and struvite-K crystals and enhanced bonding between ultrafine fly ash/ultrafine slag powder and struvite-K. As indicated by the pronounced hydration rims around the ultrafine fly ash particles, a robust bond appears to form between the ultrafine fly ash and the product, which is believed to positively impact the mechanical properties. At the same time, when ultrafine fly ash is used as a filler in MKPC and there is an early increase in the dosage of MgO and phosphate, the fast reaction bond is reduced and the volume stability is gradually improved. In the right amount, ultrafine slag powder can play a role as a filler to reduce porosity; however, if the dosage continues to increase, the ultrafine slag powder calcium ions and retarder reaction also increase, resulting in early rapid contraction of the MKPC hardening reaction and increased volume shrinkage. With the addition of 50 wt% UFA, the amount of reaction products formed in the MKPC pastes was not sufficient to effectively bind all of the UFA particles to the unreacted MgO. Hence, the added 50 wt% UFA slightly reduced the strength of the MKPC pastes, especially at early ages.

Densification of the cement paste has a great influence on the mechanical properties of MKPC. Introducing 30 wt% ultrafine fly ash (UFA) and 20 wt% ultrafine slag (USL) reduces the overall porosity, yet it has varying effects on the strengths of MKPC paste over time. Specifically, adding 20 wt% ultrafine slag powder slightly reduces the compressive strength, while incorporating 30 wt% ultrafine fly ash substantially enhances the MKPC paste’s compressive strength. However, further increasing the addition of ultrafine slag powder/ultrafine fly ash to 40 wt%/50 wt% raises the total porosity of the MKPC paste, leading to significant decreases in compressive strengths. This suggests that factors other than microstructure (pore structure) can affect the mechanical properties of MKPC. It is possible that the glass-like aluminosilicate fractions in UFA react in the aqueous phosphoric acid solution of the MKPC–UFA system, leading to the formation of secondary amorphous products with silico-phosphates [20]. These emergent products amalgamate with struvite-K, thereby enhancing the integrity of the entire hardened body and consequently augmenting its mechanical robustness. Additionally, the morphological attributes of struvite-K may contribute to this phenomenon. The development of a cohesive structure, facilitated by the entanglement and linkage of struvite-K particles, is pivotal for the augmentation of mechanical properties [57]. Higher MKPC specimen strengths are also typically associated with improved crack resistance. First, the high-strength cement design results in a denser, more homogeneous paste matrix that provides better crack resistance. This dense matrix reduces the permeability of the cement sample, limiting the entry of water, chemicals, and other harmful substances that can cause cracking over time. In addition, the MKPC hydration process is an important factor in strength development and crack resistance enhancement. During the cement mixing process, a properly designed MKPC specimen promotes repeated hydration of the internal particles, resulting in a more cohesive and durable slurry that is less likely to crack. Tchakouté [59] synthesized phosphate metakaolin-based geopolymer cementitious material in a laboratory setting, achieving a strength of 54 MPa at 28 d post-formation, by initiating a reaction between metakaolin and a phosphoric acid solution. Their findings indicate that the dispersion of berlinite within the matrix, serving to fortify the matrix, was a primary factor behind the observed strength enhancement. Although such berlinite inclusions were not detected in the MKPC paste incorporating UFA, the formation of a fortified hybrid microstructure was nonetheless observed (Figure 11a). Moreover, the intrinsic physical properties of struvite-K may also influence the overall strength of MKPC pastes, although this aspect has not been explored within the current research. In summary, the long-term compressive strength of MKPC mixed with ultrafine fly ash is higher than that of MKPC mixed with ultrafine slag powder observed after a long curing time. This is mainly attributed to the activation degree and structure of ultrafine fly ash in the MKPC system, which causes (I) the aluminum and silicon elements present in the struvite-K to contribute to the formation of adhesive and reinforced structures, (II) the formation of excess secondary amorphous aluminosilicate phosphate products beyond struvite-K, and (III) denser interfacial structures that are formed between ultrafine fly ash particles and crystals.

## 4. Conclusions

The effects of ultrafine fly ash and ultrafine slag powder on the microstructure and properties of MKPC were studied. From our experimental work, the following main conclusions can be drawn:

1. Whether ultrafine fly ash (UFA) or ultrafine slag powder (USL) is added, the primary hydration phase formed in MKPC paste is still struvite-K. Both ultrafine fly ash (UFA) and ultrafine slag powder (USL) contribute aluminosilicate fractions to the acid–base hydration reactions of MKPC. Consequently, aluminum and silicon are integrated into the struvite-K crystals in the cured MKPC pastes containing either of the two ultrafine mineral admixtures. However, in the MKPC paste containing ultrafine slag powder, Al, Si, and even Ca elements are present in the resulting struvite-K product.

2. The addition of ultrafine slag powder initially increases the compressive strength of the MKPC paste at early ages, but it does not perform as well as the control paste at later ages. In contrast, the positive effect of ultrafine fly ash on the strength of MKPC is clearly observed at later testing ages. At 28 d of curing, the compressive strength of MKPC containing 30 wt% ultrafine fly ash was increased by 17.1% compared to the control group. Notably, at 180 days of curing, the MKPC mortar with 50 wt% ultrafine fly ash achieved a compressive strength similar to that of the control paste. Overall, the MKPC phases with ultrafine fly ash show better compressive strength development compared to those with ultrafine slag powder. The incorporation of 30 wt% ultrafine fly ash enhances compressive strengths across all testing ages. This is due to the formation of a stronger microstructure in MKPC mixed with ultrafine fly ash and denser interfaces between the hydration products and ultrafine fly ash particles.

3. Regardless of the addition of ultrafine fly ash or ultrafine slag powder, the MKPC mortar presents the phenomenon of autogenous shrinkage under the rapid reaction of the paste in the early stage. The addition of ultrafine fly ash effectively improves the autogenous shrinkage of MKPC mortar in the early stage, while the addition of ultrafine slag powder significantly increases the early autogenous shrinkage of MKPC. It is noteworthy that the final volumetric autogenous shrinkage of MKPC mortar with 50 wt% UFA addition was reduced by as much as 80.8% compared to the control mortar. The German Micro-Epsilon displacement sensor technology is not only applicable to the displacement measurement of cement and other cementitious materials but also can be applied to sensing the deformation of products in a fixed space in order to improve the quality and safety of actual products; however, there are some limitations, such as the sensor accuracy, environmental adaptability, and cost constraints. Further research can be devoted to addressing these limitations to further advance the development and application of the technology.

4. The addition of 30 wt% ultrafine fly ash and 20 wt% ultrafine slag powder effectively refines the paste structure of MKPC, reducing the total porosity, particularly in the pore volume within the diameter range of 0.1~10 μm, thereby enhancing internal bonding compactness. For example, the MKPC with the addition of 20 wt% ultrafine slag powder had fewer pores with pore sizes from 0.1~10 μm at 28 d compared to the control, with a porosity of 10.7%. In comparison to MKPC containing ultrafine slag powder, MKPC supplemented with ultrafine fly ash had a visibly denser structure with less porosity, especially in the 30 wt% addition. The total porosity of UF30 is 4.2%, which is less than the total porosity of 10.7% for US20 at 28 days. However, the total porosity of MKPC also increases with the elevated dosage of ultrafine admixture. Moreover, with the addition of an appropriate quantity of ultrafine admixture, particularly ultrafine fly ash, amorphous aluminosilicate phosphate products intercalate with struvite, promoting the development of long-term strength properties in MKPC.

## Figures and Tables

**Table 1:** Chemical compositions of raw materials/wt%.

Raw Material	MgO	CaO	Fe_2_O_3_	Al_2_O_3_	SiO_2_	LOI
Magnesia	95.39	1.57	0.73	0.17	1.54	0.32
Ultrafine fly ash	0.92	4.07	4.48	31.65	50.53	2.77
Ultrafine slag powder	8.82	41.51	0.63	11.89	33.39	−0.28

**Table 2:** The proportions of alkaline components in MKPC/wt%.

Mix ID	MgO	UFA/USL
Control	100	0
UF20/US20	80	20
UF30/US30	70	30
UF40/US40	60	40
UF50/US50	50	50

UFA: ultrafine fly ash, USL: ultrafine slag powder.

## Data Availability

The original contributions presented in the study are included in the article, further inquiries can be directed to the corresponding author.
